# Development of 3D-Stacked 1Megapixel Dual-Time-Gated SPAD Image Sensor with Simultaneous Dual Image Output Architecture for Efficient Sensor Fusion †

**DOI:** 10.3390/s25216563

**Published:** 2025-10-24

**Authors:** Kazuma Chida, Kazuhiro Morimoto, Naoki Isoda, Hiroshi Sekine, Tomoya Sasago, Yu Maehashi, Satoru Mikajiri, Kenzo Tojima, Mahito Shinohara, Ayman T. Abdelghafar, Hiroyuki Tsuchiya, Kazuma Inoue, Satoshi Omodani, Alice Ehara, Junji Iwata, Tetsuya Itano, Yasushi Matsuno, Katsuhito Sakurai, Takeshi Ichikawa

**Affiliations:** Canon Inc., 70-1, Yanagi-cho, Saiwai-ku, Kawasaki 212-8602, Kanagawa, Japan

**Keywords:** SPAD, dual-time-gated, sensor fusion, RGB-D, HDR

## Abstract

Sensor fusion is crucial in numerous imaging and sensing applications. Integrating data from multiple sensors with different field-of-view, resolution, and frame timing poses substantial computational overhead. Time-gated single-photon avalanche diode (SPAD) image sensors have been developed to support multiple sensing modalities and mitigate this issue, but mismatched frame timing remains a challenge. Dual-time-gated SPAD image sensors, which can capture dual images simultaneously, have also been developed. However, the reported sensors suffered from medium-to-large pixel pitch, limited resolution, and inability to independently control the exposure time of the dual images, which restricts their applicability. In this paper, we introduce a 5 µm-pitch, 3D-backside-illuminated (BSI) 1Megapixel dual-time-gated SPAD image sensor enabling a simultaneous output of dual images. The developed SPAD image sensor is verified to operate as an RGB-Depth (RGB-D) sensor without complex image alignment. In addition, a novel high dynamic range (HDR) technique, utilizing pileup effect with two parallel in-pixel memories, is validated for dynamic range extension in 2D imaging, achieving a dynamic range of 119.5 dB. The proposed architecture provides dual image output with the same field-of-view, resolution, and frame timing, and is promising for efficient sensor fusion.

## 1. Introduction

Sensor fusion plays an important role in ensuring the robustness of vision systems across a wide range of applications, including automotive, AR/VR, mobile phones, and machine vision applications. The integration of data from multiple sensors with mismatched fields of view, resolutions, and frame timings incurs a substantial computational cost. To overcome this challenge, researchers have developed multi-functional sensors capable of operating with a monocular configuration.

One of the major topics of sensor fusion is RGB-Depth (RGB-D) imaging. Commercially available systems have utilized a combination of an RGB camera and a time-of-flight (ToF) camera. This approach requires complex post-processing to generate RGB-D images. Additionally, differences in the field of view between the two cameras can cause occlusion artifacts. To address these challenges, researchers have proposed several approaches [1,2,3,4]. In [1,2], a monolithic sensor integrates RGB and ToF pixels within the same focal plane, achieving the same field of view and frame timing for both an RGB image and a depth image, whereas their resolutions differ. A method that alternately captures RGB and ToF images [3] satisfies the spatial factors but causes mismatched frame timing. Consequently, these approaches reduce spatial or temporal fill factor for RGB and ToF images, and limit signal-to-noise ratio (SNR) and depth precision. Stacking organic photoconductive RGB film pixels on silicon indirect-ToF (i-ToF) pixels [4] improves both spatial and temporal fill factors, yet the resolution remains different. Therefore, despite these advancements, the image alignment process is still required due to differences in resolution and frame timing.

Another important topic is the dynamic range extension in 2D imaging. High dynamic range (HDR) 2D imaging typically requires combining multiple images of different exposure times captured at different frame timings and then aligning them. To ensure the same frame timing, one applicable technique is to utilize multiple parallel in-pixel memories or nodes in CMOS [5] and single-photon avalanche diode (SPAD) [6] image sensors. Yet, further improvements in HDR performance are still needed.

Recently, the SPAD image sensor has attracted attention for its natively digital operation principle and high temporal resolution [7,8,9]. Utilizing these features, time-gated SPAD image sensors, which enable selective photon detection within the nanosecond exposure window, have been developed [10,11,12,13]. These sensors support multiple sensing modalities, including 2D imaging and 3D ToF sensing with a monocular configuration. Despite these efforts, the mismatched frame timing of multiple images still remains when capturing moving objects. Therefore, integrating images, such as generating a point cloud or an HDR image, requires some extra temporal alignment. Regarding this issue, dual-time-gated SPAD image sensors capable of capturing dual images have been developed [14,15], which helps mitigate the frame timing problem. However, relevant applications of the dual-time-gated SPAD image sensors with low-to-medium resolution and medium-to-large pixel pitch have been mostly limited to bio-imaging and spectroscopy [14,15,16]. The sensor described in [14] has 15 kpixel and employs a configuration where odd and even columns can be assigned different time gates. The effective resolution for each image is reduced by half when capturing two images at different time gates. The sensor developed in [15], with 250 kpixel and a 16.38 µm pitch, offers medium resolution, yet higher resolution is still required for imaging and sensing applications. Additionally, its inability to independently adjust exposure windows for dual images restricts the flexibility of exposure settings.

We have developed a 5 µm-pitch, 3D-backside-illuminated (BSI) 1Megapixel dual-time-gated color SPAD image sensor, enabling the simultaneous output of dual images for efficient sensor fusion [17]. The dual images are captured simultaneously from a single sensor, and these images have the same field of view, resolution, and frame timing. Moreover, the exposure time of dual images can be independently controlled. The sensor is verified to operate as an RGB-D sensor without complex image alignment. In addition, a novel HDR technique utilizing the pileup effect is demonstrated for dynamic range extension in 2D imaging. In this paper, we describe the details of the architecture, operations, time-gating performance, and imaging results of the 5 µm-pitch, 1Megapixel dual-time-gated SPAD image sensor.

## 2. Materials and Methods

### 2.1. Sensor Architecture

**Figure 1:**
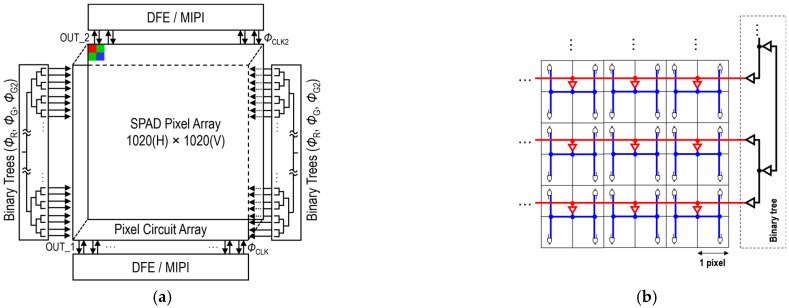

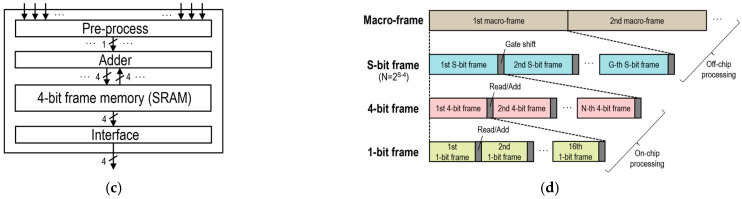
(**a**) Sensor block diagram. (**b**) Binary clock tree configuration. (**c**) DFE/MIPI block. (**d**) Frame timing diagram.

Figure 1a shows the sensor block diagram. The sensor comprises a SPAD pixel array, binary clock trees, and digital front end (DFE)/mobile industry processor interface (MIPI) blocks. A pixel array consists of a 1020 × 1020 3D-BSI SPAD array, with each photodiode connected to a corresponding pixel circuit. In order to maintain a fill factor of nearly 100% and achieve a pixel pitch of 5 μm, a charge focusing SPAD pixel is utilized [18]. The photodiode design within each pixel is based on our previous work [13], except for the addition of an on-chip color filter. The pixel circuit and peripheral circuit have been redesigned to accommodate in-pixel dual memory and two sets of readout circuits, enabling the simultaneous capture of two images. Dual gate windows can be controlled by recharge and gating pulses (Φ_R_, Φ_G_, and Φ_G2_), distributed through two binary trees on the left and right sides of the pixel array. Figure 1b illustrates the binary clock tree configuration. The global clock pulse is distributed from a buffer centrally located for each group of four pixels to minimize propagation delay skew among the pixels while reducing wiring resources. Figure 1c shows the configuration of the DFE/MIPI block. The DFE/MIPI block consists of a pre-process block, an adder, a SRAM-based 4-bit frame memory, and MIPI interfaces. The pixel array outputs a stream of 1-bit frames to two sets of DFE/MIPI blocks, which are summed to a 4-bit frame at a frame memory for efficient data compression. 4-bit frames are then transferred to an external FPGA via MIPI interface at 1314.7 fps. Figure 1d shows the frame timing diagram for an operation mode in which gate windows are shifted. The successive 4-bit frames are further aggregated into a higher bit-depth frame through off-chip processing, such as on an FPGA or a PC. In this figure, N 4-bit frames are summed to generate an S-bit frame, where N and S satisfy the relationship N = 2^S−4^. Additionally, the diagram shows an example in which dual gate windows are shifted G times to acquire a macro-frame. G and N can be tuned based on the frame rate and bit-depth requirements of various use cases. The external FPGA generates synchronized trigger pulses for laser emission, as well as Φ_R_, Φ_G_, and Φ_G2_ pulses, with the rise and fall edge timings individually adjustable to a resolution of 100 ps.

### 2.2. Pixel Architecture and Operations

**Figure 2:**
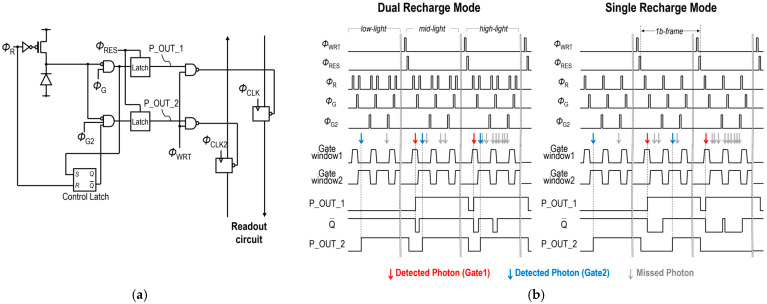

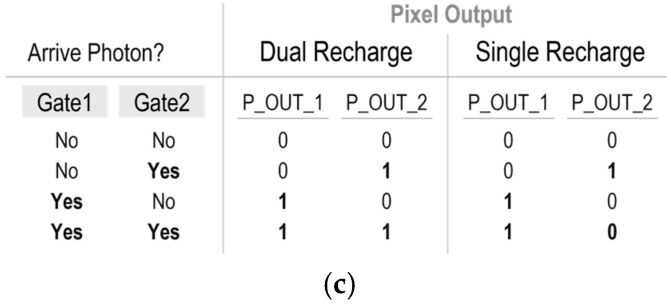
(**a**) Pixel circuit diagram. (**b**) Pixel timing diagram in dual recharge mode (**left**) and single recharge mode (**right**). (**c**) Relationship between the status of photon arrival and the corresponding pixel output.

Figure 2a shows a pixel circuit architecture. Each pixel has two parallel 1-bit memories for capturing two individual images. For each pixel, Φ_R_ and Φ_G_ define gate window 1 for P_OUT_1, while Φ_R_ and Φ_G2_ define gate window 2 for P_OUT_2. Each pixel also has a control latch, which is set when a photon is detected during the period of gate window 1 and reset by Φ_R_, and gate window 2 is controlled by the output signal $\bar{Q}$ from the control latch. Figure 2b shows a pixel timing diagram. This dual-time-gated SPAD image sensor can be operated in both conventional dual-recharge mode and the proposed single-recharge mode. In dual recharge mode, the second recharge operation is performed between gate window 1 and gate window 2, and the control latch is not utilized. Therefore, each gate window is independently defined, and any photons are simply detected within each gate window. In contrast, in single recharge mode, the second recharge operation is not performed, thereby activating the control latch. Therefore, photon detection in gate window 1 disables subsequent gate window 2 because $\bar{Q}$ is set to 0, and no photons will be detected in gate window 2 until the next recharge operation. This effect in single recharge mode is referred to as the pileup effect in the following discussion. Figure 2c shows the relationship between the status of photon arrival in each gate window and the corresponding pixel output. Under low-light conditions, photons mainly arrive during gate window 2, and P_OUT_2 is set to 1 in both modes. In contrast, under high-light conditions, the probability of photon arrival in both gate window 1 and gate window 2 increases. In dual recharge mode, both photons can be detected, but in single recharge mode, photons arriving within the gate window 2 cannot be detected due to the pileup effect. Therefore, single-recharge mode reduces the probability of photon detection in gate window 2 under high-light conditions.

## 3. Experimental Results

### 3.1. SPAD Pixel Performance

**Figure 3:**
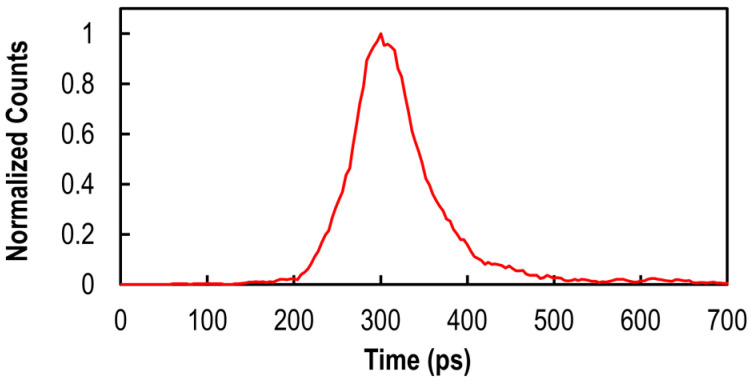
Timing jitter measurement result obtained with a 940 nm pulsed laser.

The pixel array employs a Bayer array of on-chip color filters, and the transmittance of the color filter at 940 nm is over 95% for all R, G, and B channels. The measured photon detection efficiency (PDE) at 940 nm is 23%, and the dark count rate (DCR) at 25 °C of 3.9 cps was measured. Figure 3 shows the timing jitter measurement result. The timing jitter was measured as 75 ps full-width half-maximum (FWHM) with a laser illumination wavelength of 940 nm. These characteristics are achieved while consuming 13 fC of charge per pulse. All measurements are conducted with the excess bias at 2.1 V.

### 3.2. Time-Gating Performance

**Figure 4:**
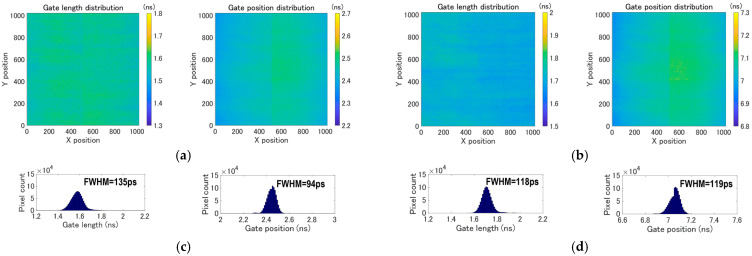
Measured gate length distribution (**left**) and gate position distribution (**right**) over 1020 × 1020 pixels at (**a**) gate window 1 and (**b**) gate window 2. Histograms for gate length (**left**) and gate position (**right**) at (**c**) gate window 1 and (**d**) gate window 2.

Figure 4 shows the time-gating performance at room temperature. To evaluate the gating performance, a laser pulsed at 10.4 MHz (wavelength: 931.8 nm, optical pulse width: ~50 ps (FWHM)) illuminates the whole pixel array. The dual gate windows are shifted with respect to the laser trigger by steps of 100 ps. For each gate position, 10-bit frames are captured and used to construct gate profiles. These profiles were analyzed to obtain the spatial distributions. Figure 4a shows the spatial distribution of gate length and gate rising edge position at gate window 1, while Figure 4b shows the same measurements for gate window 2. Figure 4c shows the histograms of gate position and gate rising edge position at gate window 1, while Figure 4d shows the same measurements for gate window 2. The measured gate length variation and gate position skew over the 1020 × 1020 pixels at gate window 1 are 135 ps and 94 ps (FWHM), respectively. At gate window 2, the variation and skew are 118 ps and 119 ps (FWHM), respectively. These values include the laser pulse width. The largely symmetric distributions shown in the histograms indicate that uniform clock delivery over the array has been successfully achieved. The minimum gate lengths achieved are 1.6 ns at gate window 1 and 1.7 ns at gate window 2.

### 3.3. RGB-D Imaging Demonstration

**Figure 5:**
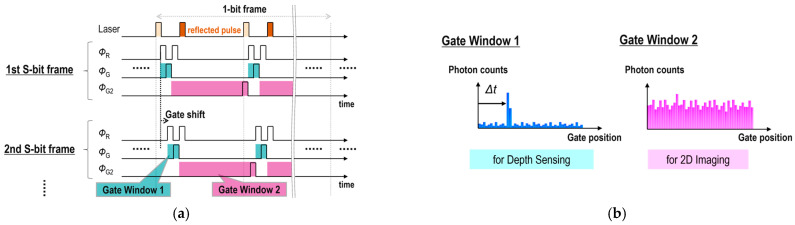
(**a**) Timing chart for RGB-D operation. (**b**) Expected photon count distribution.

Conventional RGB-D imaging approaches [1,2,3,4] have faced limitations in simultaneously achieving the same resolution, field of view, and frame timing. In contrast, RGB-D imaging using this sensor can satisfy all these capabilities. In RGB-D imaging, two in-pixel 1-bit memories are used to capture a depth image and a 2D RGB image, respectively. Figure 5a illustrates a timing chart for RGB-D operation in dual recharge mode. In RGB-D operation, gate window 1 and gate window 2 are alternately exposed in an interleaved manner, synchronized with the laser pulses, during each 1-bit frame. The timing sequence for each pixel enables capturing both a depth image and a 2D RGB image simultaneously. Gate window 1 and gate window 2 are shifted with respect to the laser pulse emission. A depth image is captured based on the ToF principle. Each 1-bit frame spans multiple laser cycles. After acquiring a multi-bit frame at a single gate position, the gate position is shifted for the next frame. Figure 5b shows the expected photon count distribution as a function of gate position. The 3D ToF image is reconstructed from multiple 2D images captured with a shifted gate window 1. The distance from the camera to the target is estimated by detecting the peak in the photon count histogram. The multi-bit 2D RGB image is obtained by accumulating 2D images captured with gate window 2.

**Figure 6:**
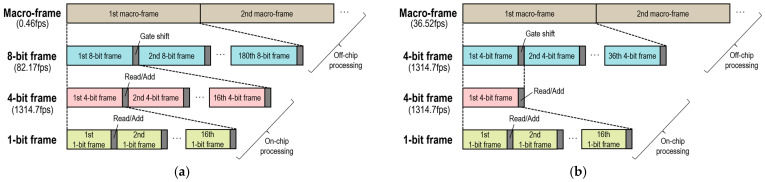
Frame timing diagrams for RGB-D imaging under (**a**) high-depth-precision and (**b**) high-frame-rate operations.

By adjusting the number of accumulated frames, the range of gate shift, and the gate shift step, various operations can be performed. Figure 6 illustrates the frame timing diagrams for RGB-D imaging, showing example settings for two operational modes: high-depth-precision operation and high-frame-rate operation. In high-depth-precision operation, the dual gate windows are shifted with respect to the laser trigger by steps of 100 ps over a range of 18 ns. As shown in Figure 6a, a single depth frame (corresponding to a macro frame in the diagram) consists of 180 gate-shifted 8-bit frames, resulting in a frame rate of 0.46 fps. Conversely, in high-frame-rate operation, each depth frame comprises 36 gate-shifted 4-bit frames, where the gate windows are shifted by 500 ps steps over the same range (Figure 6b). This operation enables RGB-D image capture at a frame rate of 36.52 fps.

**Figure 7:**
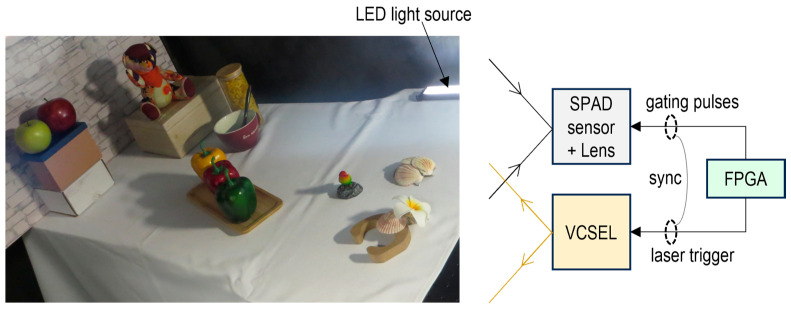
Schematic diagram of experimental setup for RGB-D imaging.

Figure 7 shows a schematic diagram of the experimental setup for RGB-D imaging. The system utilizes a 940 nm VCSEL with output power below the Class 1 safety limit and a pulse width of approximately 5 ns. The laser is pulsed at 10.4 MHz and illuminates the entire scene. The external FPGA generates the gating pulses for the SPAD image sensor and the laser trigger for the VCSEL, ensuring synchronization between them. RGB-D imaging was performed using a flash light detection and ranging (LiDAR) system configuration under LED illumination in an indoor environment with ambient light levels below 200 lux.

**Figure 8:**
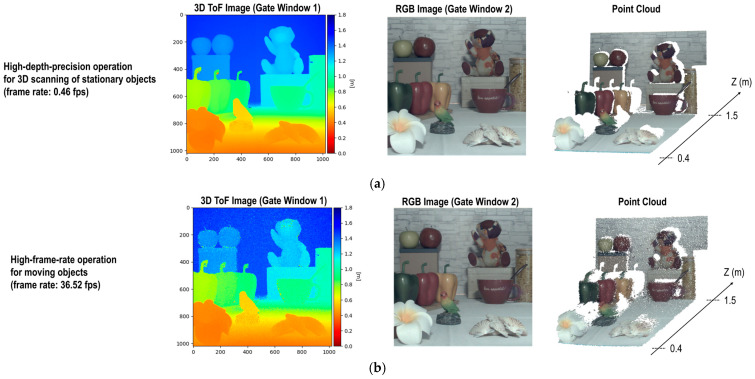
Measured 3D images, 2D RGB images, and reconstructed point clouds under (**a**) high-depth-precision and (**b**) high-frame-rate operations.

Figure 8 shows measured 3D images, 2D RGB images, and reconstructed point clouds under high-depth-precision and high-frame-rate operations. The 3D ToF image and the 2D RGB image were captured with full resolution at the same field of view and frame timing, thereby simplifying the reconstruction process of point clouds that contain both color and depth information. In addition, occlusion artifacts, which occur when using RGB and depth cameras with different field-of-view, can be suppressed as a result of the parallax-free alignment between depth and RGB images. The 3D ToF images were reconstructed using peak detection with curve fitting. Figure 8a shows the RGB-D image under high-depth-precision operation. The RGB-D image was obtained at a frame rate of 0.46 fps, which is suited for 3D scanning of stationary objects. Figure 8b illustrates the high-frame-rate RGB-D image captured at 36.52 fps. Despite the reduced depth precision, this operation is appropriate for moving objects, and the reconstructed point cloud is still reliable for some tasks, such as object detection.

The demonstrated RGB-D imaging concept simultaneously achieves three capabilities: the same resolution, field-of-view, and frame timing. A limitation, however, is that near-infrared (NIR) light cannot be filtered during RGB image capture (i.e., during gate window 2), making imaging in environments with ambient NIR illumination—such as outdoors—challenging and requiring additional image processing to improve color reproduction.

### 3.4. HDR Imaging Demonstration

**Figure 9:**
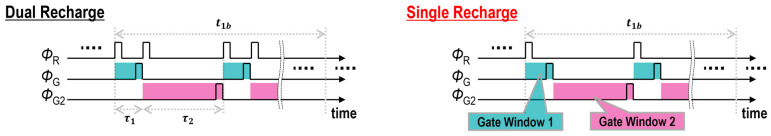
Timing chart for HDR operation in dual and single recharge modes.

The 2D HDR imaging performance of the newly proposed single-recharge mode, which utilizes the pileup effect, is compared with that of the conventional dual-recharge mode. Timing charts for HDR operation in dual and single recharge modes are shown in Figure 9. The timing charts show the sequences in a 1-bit frame, where $\tau_{1}$ and $\tau_{2}$ represent the durations of gate window 1 and gate window 2, respectively. $t_{1b}$ denotes a period of a 1-bit frame. The number of Φ_R_-Φ_G_ pairs and Φ_R_-Φ_G2_ pairs per 1-bit frame is denoted by $k_{1}$ and $k_{2}$, and these numbers also represent the number of gate window intervals within a 1-bit frame. In this experiment, the ratio between $\tau_{1}$ and $\tau_{2}$ is set to 1:10.

**Figure 10:**
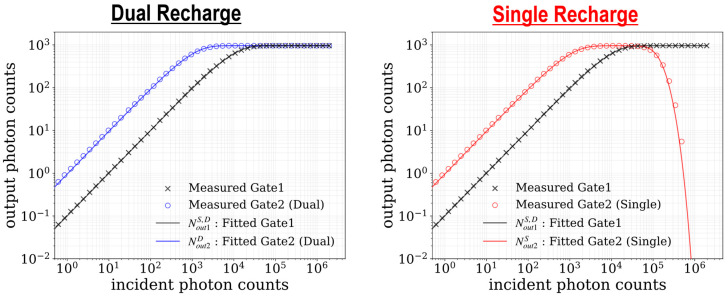
Measured and fitted output photon counts as a function of incident photon counts in dual and single recharge modes.

Figure 10 shows the measured and fitted output photon counts as a function of incident photon counts (photon response curve) in dual and single recharge modes. A 10-bit image is generated by aggregating 64 frames of 4-bit image (hereafter referred to as an aggregated frame). The saturation value of the output photon counts is 960 (15 counts × 64 frames). The solid lines in Figure 10 represent the fitted curves. In the following, the equation for obtaining the fitting curve is derived. The incident photon counts during a single gate window 1 ($H_{1}$) and incident photon counts during a single gate window 2 ($H_{2}$) are calculated using the following equations:

(1) $$H_{1}=\frac{N_{in}}{M}\cdot \frac{\tau_{1}}{t_{1b}},$$

(2) $$H_{2}=\frac{N_{in}}{M}\cdot \frac{\tau_{2}}{t_{1b}},$$

where M represents the saturation count with a value of 960, and $N_{in}$ is the incident photon counts in a single aggregated frame, which corresponds to a 10-bit frame. Under an incident photon count of $H_{1}$, the probability of no photon arrivals is $e^{-H_{1}}$, based on the Poisson process. Furthermore, the probability of no photon arrivals in gate window 1 throughout a 1-bit frame is given by ${\left(e^{-H_{1}}\right)}^{k_{1}}$. The probability that at least one photon is detected and stored in the 1-bit memory within the pixel during the 1-bit frame is expressed as $\left\{1-{\left(e^{-H_{1}}\right)}^{k_{1}}\right\}$. Since the saturation value is M, multiplying by M yields the output counts for gate window 1 in dual and single recharge modes ($N_{out1}^{S,\;D}$), as given by the following equation:

(3) $$N_{out1}^{S,\;\;D}=M\times \left\{1-{\left(e^{-H_{1}}\right)}^{k_{1}}\right\}.$$

Similarly, the output counts for gate window 2 in dual recharge mode ($N_{out2}^{D}$) is derived in the same manner and is given by the following equation:

(4) $$N_{out2}^{D}=M\times \left\{1-{\left(e^{-H_{2}}\right)}^{k_{2}}\right\}.$$

The output count for gate window 2 in single recharge mode ($N_{out2}^{S}$) is derived using a different procedure. In single-recharge mode, it is necessary to consider the photon arrival status in gate window 1 when calculating the probability of no photon arrivals in gate window 2. For each pair of gate window 1 and gate window 2 (i.e., within the duration of $\tau_{1}+\tau_{2}$, the probability that no photon is detected in gate window 2 occurs in two cases: (i) at least one photon arrives in gate window 1, or (ii) no photon arrives in either gate window 1 or gate window 2. These probabilities are expressed as $\left(1-e^{-H_{1}}\right)$ and $\left(e^{-H_{1}}\times e^{-H_{2}}\right)$, respectively. The sum of these gives the probability of no photon arrivals in gate window 2 for a single pair of gate window 1 and gate window 2. Following the same procedure as previously outlined for $N_{out1}^{S,\;D}$, $N_{out2}^{S}$ is finally calculated using the following equation:

(5) $$N_{out2}^{S}=M\times \left[1-{\left\{\left(1-e^{-H_{1}}\right)+\left(e^{-H_{1}}\times e^{-H_{2}}\right)\right\}}^{k_{2}}\right].$$

The fitted values of $k_{1}$ and $k_{2}$ are 6.4 and 6.2. The fitted curves are in good agreement with the measured output counts. In single-recharge mode, the photon response curve at gate window 2 decreases under high-light conditions due to the pileup effect as described in Section 2.2, and it enables a dynamic range extension. The dynamic range in dual recharge mode is calculated using the incident photon count at which the output count at gate window 2 reaches 99% of its saturation count and the dark random noise (DRN) measured at room temperature. In single recharge mode, it is calculated using the incident photon count at which the output count at gate window 2 reaches 1% of its saturation count, while the output count at gate window 1 is saturated. The dynamic range of 100.4 dB is measured in dual recharge mode, and 119.5 dB in single recharge mode.

**Figure 11:**
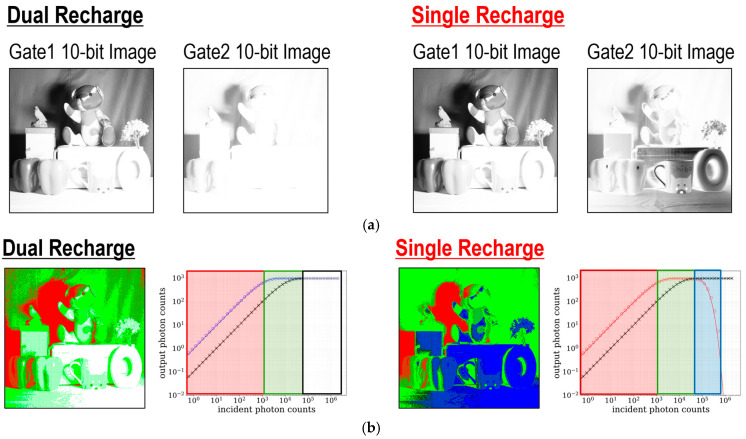
(**a**) Measured 10-bit images at gate window 1 and gate window 2. (**b**) Corresponding incident photon count maps.

Figure 11a shows the measured 10-bit images at each gate window, generated by summing up 4-bit frames. The 10-bit images captured at gate window 1 are almost identical in both modes. In contrast, the 10-bit images at gate window 2 indicate that areas saturated in dual recharge mode exhibit lower output count values in single recharge mode. The corresponding incident photon count maps are shown in Figure 11b. In the photon count map, red and green regions represent low and medium incident photon count regimes, respectively. The white region in dual recharge mode and the blue region in single recharge mode both correspond to high incident photon count regimes. The incident photon count value for each pixel can be estimated using the output photon counts at each gate window and lookup tables (LUTs) from the output count to the incident photon count. The estimation process differs between the dual-recharge mode and the single-recharge mode. In dual recharge mode, first, saturation of the output count at gate window 2 is checked. If the output count at gate window 2 is not saturated (red region), the incident photon count is reconstructed from the LUT from the output count at gate window 2 to the incident photon count. If gate window 2 is saturated but gate window 1 is not (green region), the incident photon count is derived from the LUT for gate window 1. In single recharge mode, the estimation process utilizes two separate LUTs for gate window 2: one for the low-light region (where output count increases with increasing incident photon count), and one for the high-light region (where output count decreases with increasing incident photon count). These LUTs can be generated based on photon response curves obtained through fitting. The estimation process of the incident photon counts in red and green regions follows almost the same procedure as in dual recharge mode. In the red region, where the output count values at both gate windows are unsaturated, the incident photon count is estimated using the low-light LUT for gate window 2. In the green region, the incident photon count is reconstructed from the LUT for gate window 1, similarly to the dual recharge mode. If gate window 1 is saturated but gate window 2 is not saturated (blue region), the incident photon count is estimated using the high-light LUT for gate window 2. In both modes, low-light (red) and mid-light (green) regimes are almost the same, while a gray level for the high-light regime (blue) can be represented exclusively in the single-recharge mode.

**Figure 12:**
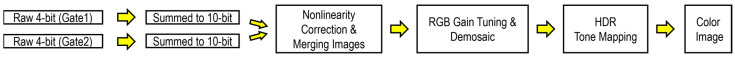
HDR image reconstruction flow.

Figure 12 illustrates the HDR image reconstruction flow based on images captured at gate window 1 and gate window 2. First, the 10-bit images are reconstructed by aggregating and summing 64 frames of 4-bit sensor outputs. Next, the incident photon counts are estimated from the output counts at gate window 1 and gate window 2 using LUTs. After color gain adjustment for white balance, the reconstructed incident photon counts are demosaiced. Finally, tone mapping is applied to the RGB values of each pixel, generating an HDR color image.

**Figure 13:**
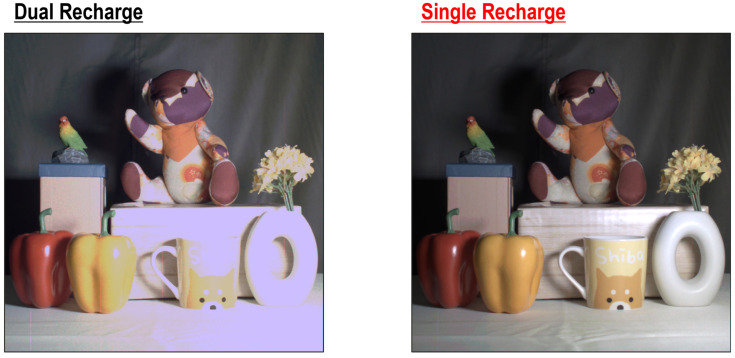
HDR color images in dual and single recharge modes.

Figure 13 shows reconstructed HDR images in dual and single recharge modes. The HDR images indicate that some parts of the scene are overexposed in dual recharge mode, while the scene is clearly visible and the color is recognized only in single recharge mode.

Table 1 shows the relationship between the bit depth of a single frame at each gate window, frame rate, and dynamic range in dual and single recharge modes. The dynamic range at 10-bit depth is obtained from the measured photon response curve, while the dynamic range for 11-bit and 12-bit depth is obtained from the photon response curve calculated using Equations (1)–(5). As shown in this table, through adjustment of the accumulation count of the 4-bit frame, both the dynamic range and frame rate can be tuned to optimize performance across various scenes and tasks.

### 3.5. Chip Power Consumption

The measured chip power consumption under saturating light is 1053 mW. The measurement was conducted while the sensor operated in dual recharge mode under high-light conditions. In this experiment, the entire pixel array was uniformly illuminated, and the output count for all pixels in each gate window reached saturation. The power consumption of the SPAD accounts for approximately 25% of the total at saturation, while the remaining power is consumed by the circuits.

## 4. Conclusions

**Figure 14:**
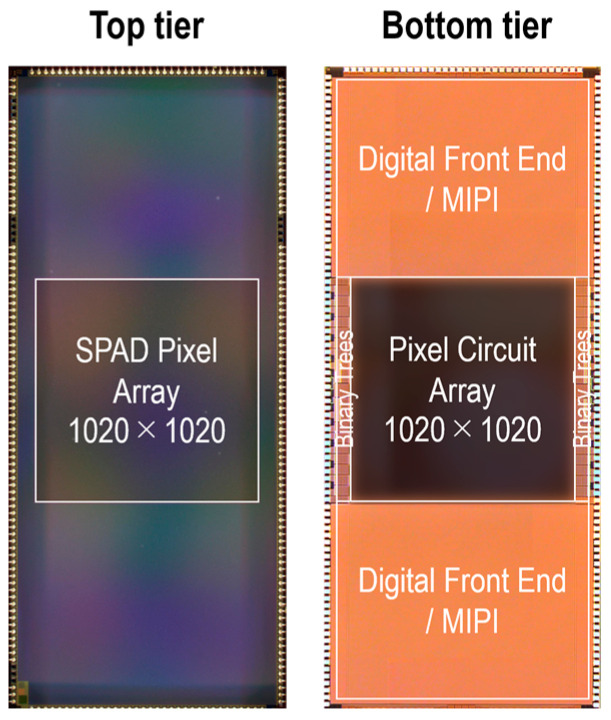
Chip micrographs.

In this paper, we present a 5 µm-pitch, 3D-BSI 1Megapixel dual-time-gated color SPAD image sensor. Figure 14 shows a chip microphotograph. The sensor can simultaneously capture individual dual images, which have the same field of view, resolution, and frame timing. The proposed sensor is verified to operate as an RGB-D sensor. In addition, a newly proposed HDR technique, utilizing the pileup effect with two parallel in-pixel memories, is validated for dynamic range extension in 2D imaging. Table 2 shows the state-of-the-art comparison for time-gated SPAD image sensors. This work demonstrates one of the smallest pixel pitches and the largest array sizes, while achieving best-in-class gating performance in terms of the minimum gate length and gate skew. The developed SPAD image sensor allows efficient sensor fusion without requiring image alignment for numerous imaging and sensing applications.

## Figures and Tables

**Table 1:** Relationship between bit depth of a frame, frame rate, and dynamic range in dual and single recharge modes.

Bit Depth of Single Frame	Frame Rate	Dynamic Range	
		Dual Recharge Mode	Single Recharge Mode
10 bit	20.54 fps	100.4 dB (calculated from measured data)	119.5 dB (calculated from measured data)
11 bit	10.27 fps	106.2 dB	125.3 dB
12 bit	5.14 fps	112.2 dB	131.3 dB

**Table 2:** State-of-the-art comparison table for time-gated SPAD image sensors.

	Perenzoni (2016) [11]	Gyongy (2018) [12]	Morimoto (2020) [10]	Wayne (2022) [15]	Morimoto (2024) [13]	This Work (Gate1/Gate2)
Process technology	350 nm HV CMOS	130 nm CIS	180 nm CMOS	180 nm CMOS	90 nm/55 nm 3D-BSI CMOS	90 nm/55 nm 3D-BSI CMOS
Die size (mm × mm)	3.42 × 3.55	5 × 5	11 × 11	9.6 × 9.7	6.3 × 10.9	6.3 × 14.8
Sensor resolution	160 × 120	256 × 256	1024 × 1000	500 × 500	1020 × 1020	1020 × 1020
Pixel pitch (µm)	15	16	9.4	16.38	5	5
Exposure modes	GS	RS/GS	RS/GS	RS	Seamless GS	Seamless GS
Frame rate (fps)	486 (5.4 bit)	100,000 (1 bit)	24,000 (1 bit)	49,800 (1 bit × 2)	1310 (4 bit)	1314.7 (4 bit × 2)
Fill factor (%)	21	61	7	10.5	~100	~100
PDE at 940 nm (%)	N/A	N/A	<0.5	N/A	23	23
Min. gate length (ns)	0.75	4.0	3.8	1.0	1.85	1.60/1.70
Gate length variation (ps)	80.2 (std. dev.) 189 (FWHM) *	N/A	120 (FWHM)	70 (std. dev.) 165 (FWHM) *	125 (FWHM)	135/118 (FWHM)
Gate skew (ps)	N/A	N/A	410 (FWHM)	109.4 (FWHM)	80 (FWHM)	94/119 (FWHM)
Power consumption at saturation (mW)	N/A	N/A	18,236 (per 1 Mpixel)	N/A	505	1053
Pixel output bit depth	5.4 bit (analog)	1 bit	1 bit	1 bit × 2 channels	4 bit	4 bit × 2 channels
Mono/Color	Monochrome	Monochrome	Monochrome	Monochrome	Monochrome	Color

* Standard deviation (σ) converted to FWHM by: $FWHM=2\sigma \sqrt{2\log2}$.

## Data Availability

The data presented in this study are available on request from the corresponding author. The data are not publicly available due to the confidentiality of the corporate activity.
